# Functional Nanocomposites: From Strategic Design to Applications

**DOI:** 10.3390/nano14231931

**Published:** 2024-11-30

**Authors:** Li Cao, Mohammed J. Meziani

**Affiliations:** 1 Department of Chemical and Materials Engineering, University of Dayton, Dayton, OH 45469, USA; 2 Department of Natural Sciences, Northwest Missouri State University, Maryville, MO 64468, USA

## 1. Introduction

Nanomaterials with one-, two-, or three-dimensional structures have exhibited superior optical, electronic, magnetic, thermal, and mechanical properties compared to their bulk material counterparts [1,2,3,4,5]. These unique properties have been extensively investigated for applications in various fields, such as light emission diodes (LEDs), solar cells, microchips, imaging agents, sensors, energy storage and conversion, and environmental protection [6,7,8,9,10]. These nanomaterials have also been widely employed as nanofillers in various polymer, ceramic, and metal matrices to fabricate advanced nanocomposites with enhanced and unique multifunctional properties [11,12,13,14,15,16,17,18]. The present Special Issue aims to further promote and expedite the research into high-performance nanocomposites to broaden their applications in a wide range of fields.

## 2. An Overview of Published Articles

Maier et al. reported the fabrication of cupric oxide nanowire (CuO NW) gas sensors on Si substrates and identified the optimum gap size, thickness, length, and width of Cu structures for efficient NW growth to bridge and electrically connect adjacent CuO structures [19]. For a film thickness of 560 nm, the best results were obtained by structures 4 × 16 μm^2^ in size with a gap size of 3 μm. These optimal parameters were then evaluated and tested directly on a sensor chip, demonstrating a better sensing response to acetylene, ethane, ethene, and propene at ppm levels than to CO and CO_2_.

Gence et al. reported the fabrication of conductive electrodes by integrating silver nanowires (AgNW) with titanium nitride (TiN) layers and then depositing them onto cellulose nanopaper and PET substrates using a low-temperature plasma-enhanced PLD technique, achieving thicknesses from 50 to 250 nm [20]. These TiN-AgNW nanocomposites demonstrated an improved electromechanical performance with minimal impact on their optical transparency and a higher stability compared to pure AgNW, TiN coatings, and ITO/PET substrates upon bending and exposure to ambient air, making them promising candidates for flexible bio-compatible electronic devices.

AlGhamdi et al. explored TiO_2_ nanoparticle-reinforced denture bases fabricated with two types of vat photopolymerization 3D printing technologies: digital light processing (DLP) and stereolithography (SLA) [21]. The mechanical properties of the 3D-printed denture bases were found to be affected by the type of material used, the nanoparticle concentration, and the time after curing. For example, incorporating TiO_2_ nanoparticles at concentrations of 1% and 2% was found to enhance flexural strength, while a 2% nanoparticle concentration resulted in a reduction in the elastic modulus and hardness of the 3D-printed nanocomposites. Their flexural strength and hardness also significantly improved with the increase in post-curing time. Gad et al. tested the effects of different denture cleansers on the color stability and surface roughness of 3D-printed denture base resins that were either unmodified or modified with ZrO_2_ nanoparticle [22]. In this study, the authors recommended using Fittydent, Corega, and water as denture cleansers while advising against the use of NaOCl. AlGhamid et al. also found that the incorporation of ZrO_2_ and SiO_2_ nanoparticles into the 3D printed resins used for provisional restoration increased their hardness without affecting their surface roughness [23]. The use of SiO_2_ resulted in only minor color changes, whereas ZrO_2_ caused noticeable color alterations, exceeding perceptibility thresholds.

Oberhausen et al. fabricated self-healing magnetic polyelectrolyte nanocomposites using different concentrations of iron oxide nanoparticles [24]. The incorporation of these superparamagnetic nanoparticles into the polymer matrix facilitated spatially resolved healing under alternating magnetic fields due to the ionic interactions between the anionic copolymer and cationically functionalized iron oxide nanoparticles.

Bandaru et al. provided a comprehensive overview of the existing stimuli-independent and -dependent methodologies used to assemble nanomaterials, along with their desired structural and functional properties and the significance of their potential applications [25]. Some of the common stimuli-independent methods highlighted included solvent evaporation, gravitational sedimentation, and oriented attachment through covalent bonding, van der Waals forces, electrostatic interactions, and cation exchange. As for the stimuli-dependent methods, common triggers included light, electric fields, magnetic fields, temperature, solvents, acid bases, metal ions, biomacromolecules, and gases. Each approach offered distinct advantages tailored to specific applications, often resulting in organized structures with precisely controlled sizes, shapes, compositions, and properties.

In their review, Singh et al. highlighted the recent advances made in the development of polymeric composites with the use of boron nitride nanosheets (BNNs) and various filler alignment strategies during composite fabrication for the creation of high in-plane, cross-plane, and isotropic thermal conductive (TC) performances [26]. The significant challenges and major opportunities for the technological application of these nanocomposites were also identified and discussed. Several key characteristics have been identified as necessary to achieve high TC performances, including the use of thinner BNNs with larger lateral sizes, fewer defects, and high crystallinity, and their effective alignment, dispersibility, and interfacial compatibility within the polymer matrix.

## 3. Conclusions

In conclusion, this Special Issue is a collection of eight articles (six original research articles and two review articles) focused on advancing the development of functional nanocomposites for novel applications. Researchers have fabricated and characterized diverse functional nanocomposites by successfully integrating nanomaterials with zero-, one-, and two-dimensional structures into different matrix materials, enabling the creation of unique structures with precisely controlled sizes, shapes, and compositions and the realization of desired functionalities and tailored properties for various applications. The targeted applications discussed in this collection include self-healing, reinforced denture bases and cleansers, gas sensor chips, conductive bio-compatible flexible electronic devices, thermal management in modern electronic devices, and others. Our Special Issue will significantly contribute to and advance the research being conducted on high-performance functional nanocomposites and lead to applications that will appeal to a broad audience within the field of nanoscience and nanotechnology.
